# A novel programmable lysozyme-based lysis system in *Pseudomonas putida* for biopolymer production

**DOI:** 10.1038/s41598-017-04741-2

**Published:** 2017-06-29

**Authors:** José Manuel Borrero-de Acuña, Cristian Hidalgo-Dumont, Nicolás Pacheco, Alex Cabrera, Ignacio Poblete-Castro

**Affiliations:** Biosystems Engineering Laboratory, Center for Bioinformatics and Integrative Biology (CBIB), Faculty of Biological Sciences, Universidad Andres Bello, Santiago, Chile

## Abstract

Cell lysis is crucial for the microbial production of industrial fatty acids, proteins, biofuels, and biopolymers. In this work, we developed a novel programmable lysis system based on the heterologous expression of lysozyme. The inducible lytic system was tested in two Gram-negative bacterial strains, namely *Escherichia coli* and *Pseudomonas putida* KT2440. Before induction, the lytic system did not significantly arrest essential physiological parameters in the recombinant *E*. *coli* (ECPi) and *P*. *putida* (JBOi) strain such as specific growth rate and biomass yield under standard growth conditions. A different scenario was observed in the recombinant JBOi strain when subjected to PHA-producing conditions, where biomass production was reduced by 25% but the mcl-PHA content was maintained at about 30% of the cell dry weight. Importantly, the genetic construct worked well under PHA-producing conditions (nitrogen-limiting phase), where more than 95% of the cell population presented membrane disruption 16 h post induction, with 75% of the total synthesized biopolymer recovered at the end of the fermentation period. In conclusion, this new lysis system circumvents traditional, costly mechanical and enzymatic cell-disrupting procedures.

## Introduction

The microbial synthesis of valuable chemicals using renewable feedstocks has opened new avenues for creating a more sustainable society. In recent decades, the field of metabolic and process engineering has made significant advances towards the efficient production of biofuels, amino acids, proteins, and biopolymers^1, 2^. However, significant challenges remain, including the crucial obstacle of fermentation and downstream processing costs^3^. This is a particular concern in the microbial production of Polyhydroxyalkanoates (PHAs), biopolymers that accumulate as inclusion bodies in the cellular cytoplasmic space under nutrient imbalance^4^. PHAs are a leading biopolymer option for replacing petroleum-based plastics given their similar mechanical and physical properties to conventional thermoplastics^5^. Commercialized PHAs are currently being produced at industrial scale by KANEKA Corporation (Japan), Biomer (Germany), Bio-on (Italy), and Meridian Inc. (USA). Among the natural PHA-producing industrial strains, *Pseudomonas* species play a key role as cell factory for the synthesis of a wide array of medium-chain-length PHAs (mcl-PHAs)^6, 7^ from various feedstocks such as waste oils^8^, by-products of the sugar, palm, and biodiesel industry^9, 10^. This is due to the high metabolic versatility displayed by *Pseudomonas* ^11^ that can cope with the adverse conditions imposed by the substrates used as carbon sources, where some of them are toxic, or even lethal, to other microbial industrial strains like *E*. *coli* and *S*. *cereviseae*. In recent years, glycerol has emerged as a promising carbon substrate for industrial biotechnological applications given its low cost, high degree of reduction, and increased supply by the biodiesel industry^12^. *Pseudomonas putida* strains has recently proved to be suitable for converting raw and pure glycerol into mcl-PHAs in batch^9, 13^, Fed-batch^10^, and chemostat cultures^14^. Under nitrogen limiting conditions, *P*. *putida* KT2440 amassed 34% of its cell dry weight (CDW) as mcl-PHA, and a final biopolymer titer of 1.4 (g/L) on 30 (g/L) raw glycerol after 72 h of cultivation^9^. In a high-cell-density Fed-batch process, *P*. *putida* GO16 reached a mcl-PHA yield of 6 (g/L) and 33% of its CDW as PHA, using raw glycerol as the only carbon substrate^10^. Traditional methods for PHA recovery from the cell involve hydrolytic enzymes, sonication, high temperatures, and solvent/detergent reagents^15^. To achieve cell lysis during cell growth, several research groups have constructed various genetic systems based on inducible promoters that result in the production of holin and endolysin proteins (HEPs)^16–19^. Bacteriophages produce these proteins to achieve peptidoglycan-degrading activity of bacterial host cell walls, with the final aim of exiting the cell^20^. The HEP phage lysis system can be used to extract lipids for biofuel production in *Synechocystis* sp., in addition to uses in protein and PHAs recovery in *Escherichia coli* ^19, 21^, *Pseudomonas putida* ^17^, *Halomonas campinensis* ^22^, and *Bacillus megaterium* ^18^. Nevertheless, some drawbacks remain to be addressed, especially for Gram-negative bacteria, (i) the titer of the target product is rather low, and most importantly (ii) the inducible lysis system works inefficiently under biopolymer-producing conditions^17^.

In this study, we constructed a genetic autolysis system in *P*. *putida* KT2440, a natural PHA-producing strain that can be activated at different phases of growth of the bacterial culture. Upon induction, the inducible system synthesizes lysozyme, which is widely used to disrupt the bacterial cell wall. Lysozyme is an enzyme (N-acetylmuramide glycanhydrolase) that hydrolyzes the 1,4-beta-linkages between N-acetylmuramic acid and N-acetyl-D-glucosamine residues in a peptidoglycan. Hydrolysis destabilizes the bacterial cell wall, instigates an osmotic imbalance, and, finally, results in cell lysis^23^. Lysozyme is abundant in various secretory environments, such as in saliva, mucus, and tears, among others. As part of the human immune system, this enzyme is extremely efficient against Gram-positive pathogens such as *Bacillus* and *Streptococcus* ^24^. Taking advantage of this efficiency, we constructed an *m*-toluic acid-inducing system to trigger the expression of the gene encoding for the C-type lysozyme of *Gallus gallus*. As this enzyme acts on the peptidoglycan layer, which is located in the periplasm of Gram-negative organisms, we sought a secretion system that would ensure the translocation of the produced recombinant lysozyme into the periplasmic space of the cell, thus resulting in membrane damage of the cell. To this end, a signal peptide (SP) described for a naturally secreted protein in *Pseudomonas stutzeri* was fused at the N-terminus of lysozyme. This resulted in high-yield cell disruption and the recovery of most synthesized biopolymers at the end of the fermentation period under nitrogen-limiting conditions in batch cultures.

## Results

### Construction of the lytic system in *Escherichia coli* and *Pseudomonas putida* strain

**Figure 1:**
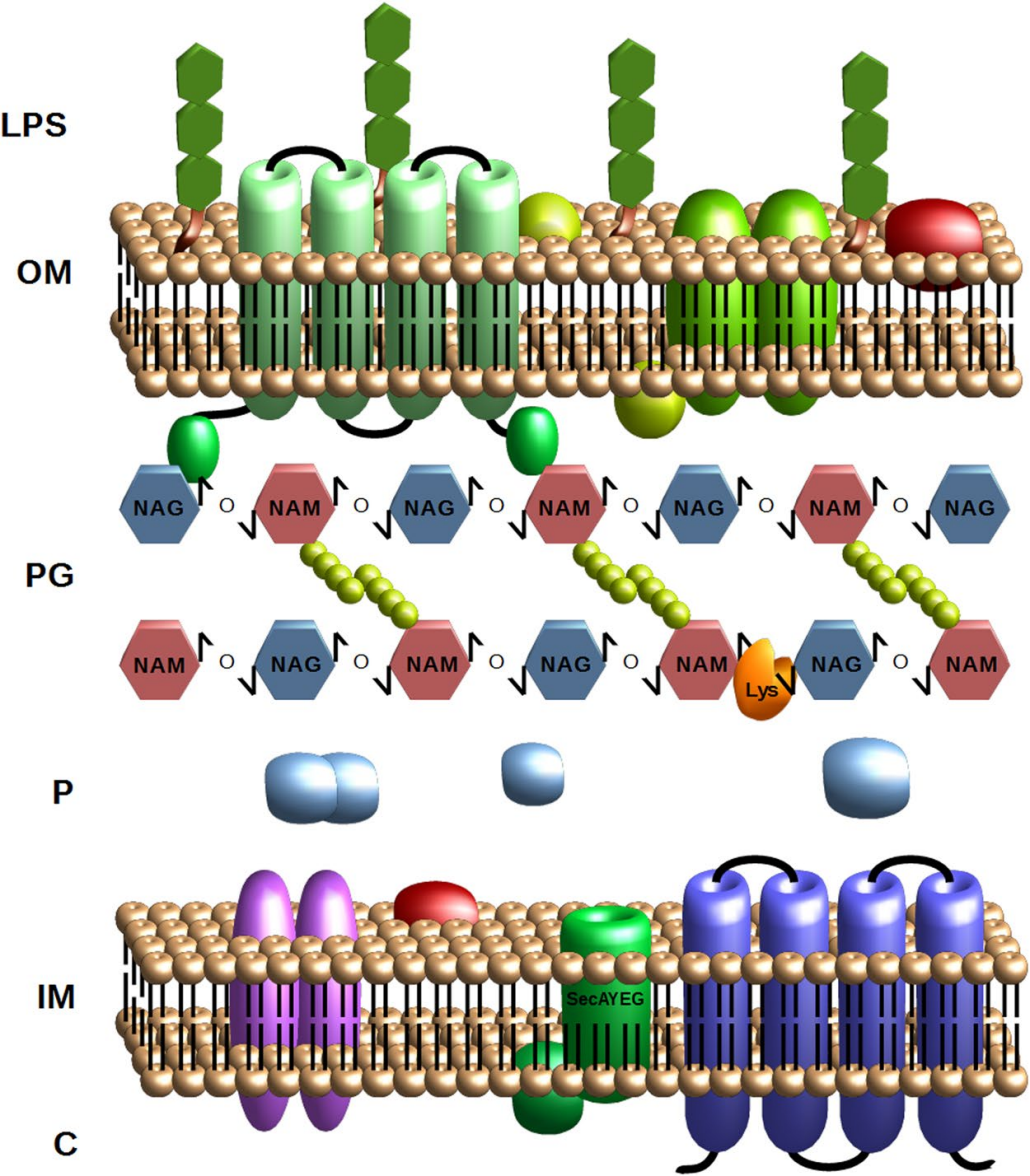
Generic representation of the Gram-negative subcellular fractions. The lysozyme catalytic action is illustrated. LPS = lipopolysaccharide; OM = outer membrane; PG = peptidoglycan; P = periplasm; IM = inner membrane; and C = cytoplasm.

**Figure 2:**
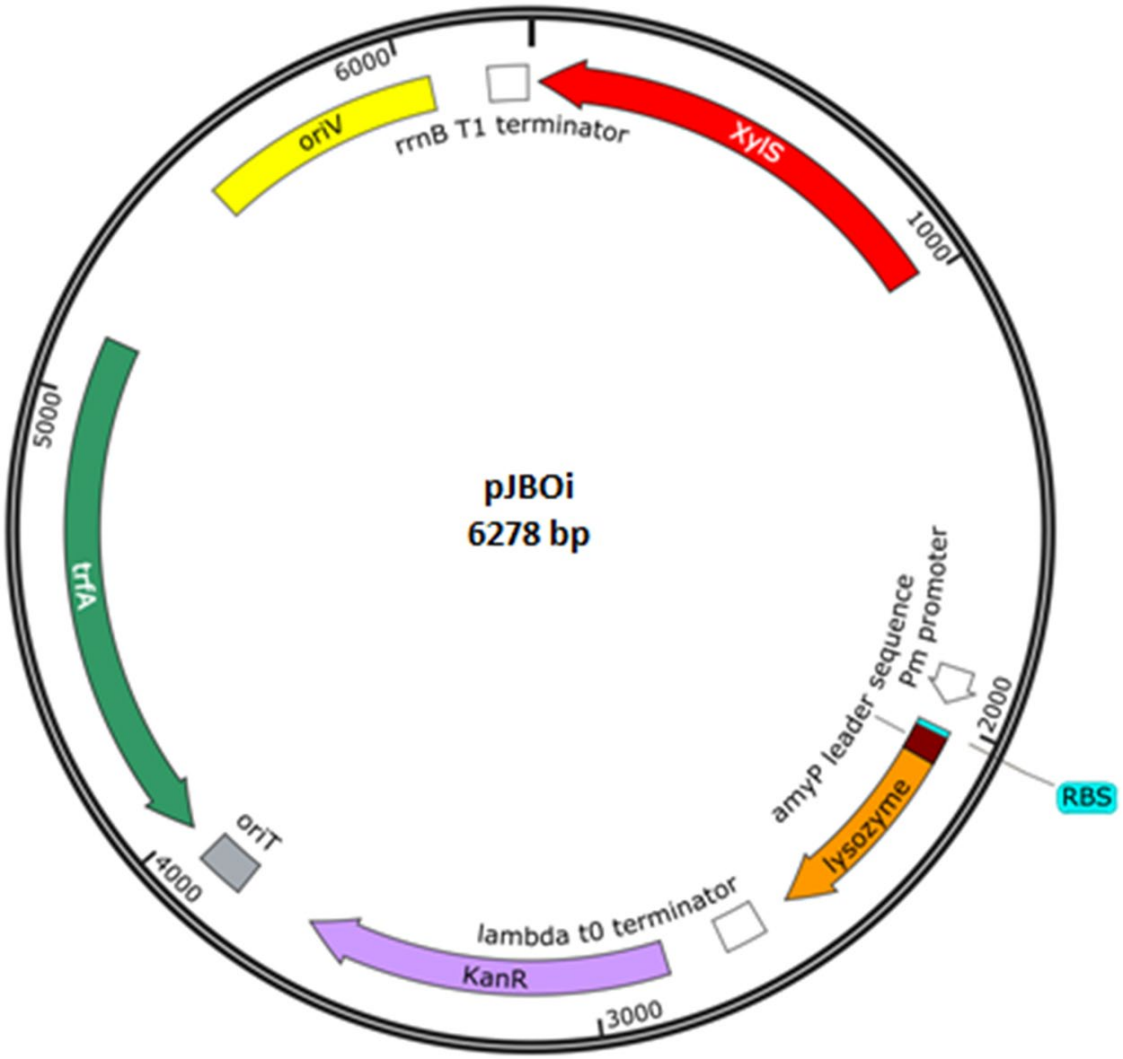
Illustration of the pSEVA228 expression plasmid employed in this study. The different specific features of this plasmid are outlined.

A novel genetic strategy for cell autolysis was designed based on the inducible expression of the peptidoglycan-disrupting lysozyme, which is used worldwide for cell breakage. This enzyme catalyzes the 1,4-beta-linkages between N-acetylmuramic acid and N-acetyl-D-glucosamine residues, which compose the peptidoglycan layer (Fig. 1). Hydrolysis of these bonds results in the destabilization of the bacterium membrane since the peptidoglycan confers mechanical resistance to the cell. Therefore, we hypothesized that catalytic actions of this enzyme in the subcellular periplasm compartment would lead to cell disruption, thus exposing the products of interest for full recovery. This lysozyme was translocated into the periplasm by flanking the lysozyme gene with a *P*. *stutzeri* N-terminal SP. The lysozyme C precursor sequence of *G*. *gallus* (NCBI: NP_990612.1) was thus fused at the amino terminus to the 63 nucleotides constituting the leader sequence of the Glucan 1,4-alpha-maltotetraohydrolase of *P*. *stutzeri* [Uniprot: P13507 (AMT4_PSEST)]. The protein is secreted to the outside of this bacterium through the recognition of the first 21 N-terminal amino acids, which are ultimately cleaved after translocation^25^. To construct this genetic circuit, the nucleotide sequence of the lysozyme C precursor gene lacking a start codon was purchased and synthesized (Integrated DNA Technologies, Inc., IA, USA). Then, the sequence was flanked by the aforementioned leader sequenced, and the *EcoR*I and *Xba*I restriction sites were included at the N- and C-termini, respectively. This construction, termed SP-lysozyme, was inserted into the pIDTSmart plasmid, which harbors an ampicillin resistance cassette (Integrated DNA Technologies, Inc., IA, USA). The resulting recombinant strain was termed *E*. *coli dam* ^−^/*dcm* ^−^ pIDTSmart-SP-lysozyme (Table 1). The genetic SP-lysozyme entity was further cloned into a pSEVA228 *EcoR*I-*Xba*I digested plasmid inducible by the addition of toluic acid^26, 27^ (Fig. 2). This final construct, termed pJBOi, was finally introduced into *E*. *coli* Dh5α and *P*. *putida* KT2440, termed the ECPi and JBOi strain, respectively (Table 1). Both recombinant strains were subsequently tested for cell self-disruption under standard growth conditions and in the case of JBOi under PHA producing conditions.

**Table 1:** Strains and plasmid used in this study.

Strain or plasmid	Special features	Source
**Bacterial strains**		
***Escherichia coli***		
*dam* ^−^/*dcm* ^−^	Genotype: *ara-14 leuB6 fhuA31 lacY1 tsx78 glnV44 galK2 galT22 mcrA dcm-6 hisG4 rfbD1 R*(*zgb210*::*Tn10*) Tet^S^ *endA1 rspL136* (Sm^R^) *dam13*::*Tn9* (Cm^R^) *xylA-5 mtl-1 thi-1 mcrB1 hsdR2*. Strain deficient in Dam and Dcm methylation	New England Biolabs, Ipswich, USA
*dam* ^−^/*dcm* ^−^ pIDTSmart-SP-lysozyme	*E*. *coli dam* ^−^/*dcm* ^−^ harboring the SP-lysozyme expression system in pIDTSmart	This study
*dam* ^−^/*dcm* ^–^ -pJBOi	*E*. *coli dam* ^−^/*dcm* ^−^ bearing the SP-lysozyme expression system in pSEVA228	This study
*E*. *coli* DH5alpha	Wild type strain	Biomedal, Spain
ECPi	*E*. *coli* harboring the SP-lysozyme expression system in pSEVA228	This study
***Pseudomonas putida***		
KT2440	Wild type strain	DSM 6125, DSMZ, Germany
JBOi	KT2440 harboring the SP-lysozyme expression system in pSEVA228	This study
**Plasmids**		
**pSEVA plasmid**		
pJBOi	pSEVA228 bearing the SP-lysozyme expression system. *xylS*-Pm (inducible by 3-methylbenzoate/toluic acid), Km^R^, RK2 replication origin	This study
**IDT plasmid**		
pIDTSmart-SP-lysozyme	pIDTSmart harboring the SP-lysozyme expression system. Ap^R^, pMB1-type ColE1 replication origin	Integrated DNA Technologies, Inc., Iowa, USA

Ap^R^ = Ampicillin resistance; Km^R^ = Kanamycin resistance; Sm^R^ = Streptomycin resistance; Cm^R^ = Chloramphenicol resistance.

### Growth characterization and lytic response in recombinant *E*. *coli* and *P*. *putida* strains under standard growth conditions

**Figure 3:**
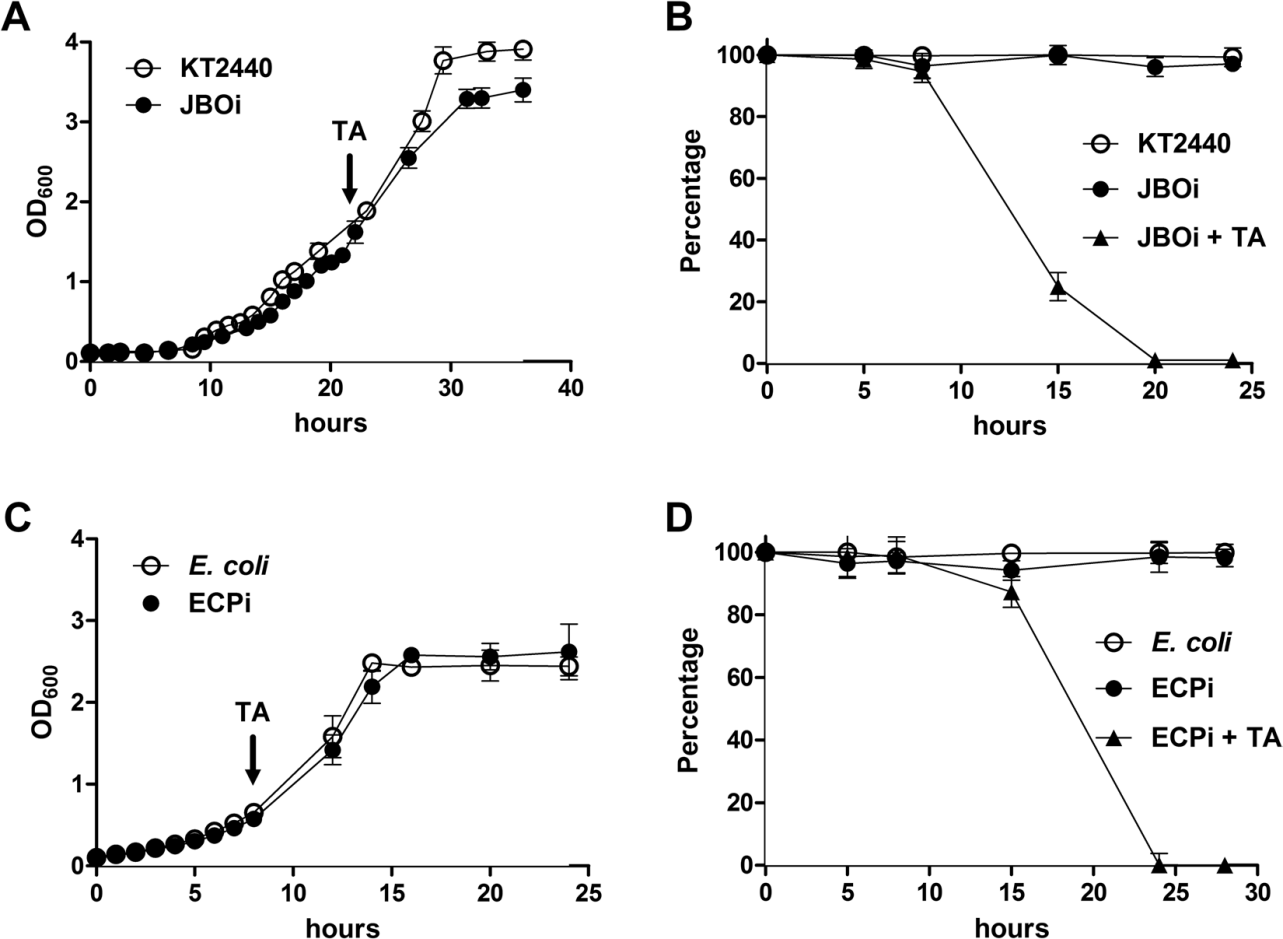
Growth profile of *P*. *putida* KT2440 and JBOi strain (**A**) and *E*. *coli* and ECPi strain (**C**) in minimal medium supplemented with 4 (g/L) glycerol. Cell lysis response of *P*. *putida* KT2440 and the engineered JBOi strain (**B**), and *E*. *coli* and ECPi strain (**D**). Arrows indicates addition of 5 mM of *m*-toluic acid (TA).

The growth pattern and lytic response in *E*. *coli* and *P*. *putida* strains harboring the SP-lysozyme system under standard growth conditions were assessed in minimal medium supplemented with 4 (g/L) glycerol, in batch cultures (see Methods). The specific growth rate of the ECPi and JBOi strain was 0.22 and 0.17 (1/h), respectively. Those values were slightly lower than that displayed by the parental *E*. *coli* and KT2440 strain (0.23 and 0.19 (1/h), respectively), so both engineered strains showed very similar growth behaviors and biomass yield compared to the parental strain (Fig. 3A,C). Using the same growth conditions described above, the lytic response of the recombinant JBOi strain growing on glycerol was assessed in batch cultures, where the addition of toluic acid (5 mM) was performed in the middle of the exponential growth phase (at 24 h of cultivation) resulting in a diminished cell counts 10 h post induction (Fig. 3B). Approximately 10% of the cell population died each hour, leaving ~1% of live cells 20 h after induction. A different scenario was observed for the recombinant ECPi strain, as cells showed no lag-phase thus, the lytic system was induced at 8 h after the start of the culture where a reduction of 15% of the viable cells can be observed at 15 h after induction. Overall, the lysozyme expression system efficiently provoked cell lysis, reaching also 1% of cell viability at 25 h in the course of the fermentation (Fig. 3D).

### Cell lysis and mcl-PHA production in the recombinant *P*. *putida* strain

**Figure 4:**
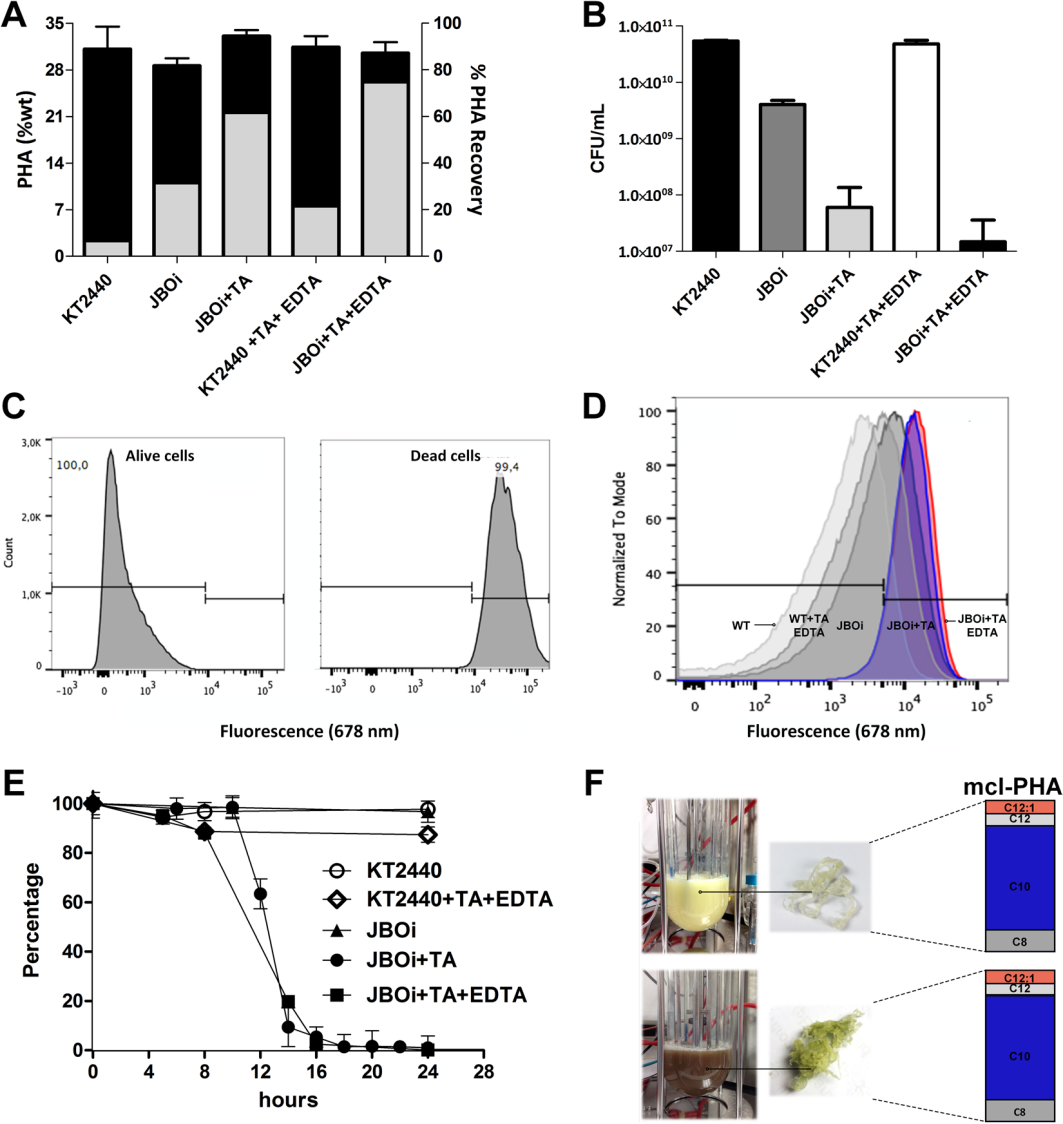
PHA yield and recovery in the recombinant and the wild type *P*. *putida* strain (**A**), CFU counts (**B**) and lysis response via FACS (**C**,**D**) after 96 h of cultivation under PHA-producing conditions in shake flask cultures. Dynamic cell lysis response of the recombinant and the wild type *P*. *putida* strain upon induction at 72 h incubation (**E**), and PHA synthesis in bioreactors showing the final monomer composition (**F**).

We next challenged the recombinant JBOi and the wild type KT2440 strain to PHA producing conditions. To do so, we increased the amount of glycerol from 4 to 30 g/L in the batch cultures. This condition triggers nitrogen limitation 16 h after the start of the culture, where the PHA accumulation stage begins. In terms of PHA productivity, the wild-type KT2440 strain amassed 33% of the cell dry weight as PHA after 72 h of incubation (Fig. 4A). The non-induced recombinant strain JBOi also accumulated 30% of the cell dry weight as PHA, but total biomass formation, in terms of CDW (g/L) and CFU counts, was reduced nearly 25% in comparison to the parental *P*. *putida* KT2440 strain (Fig. 4B). To test whether the lytic system works at the stationary phase, filtered toluic acid (5 mM) was added at 72 h to the fermentation broth, when PHA accumulation was at its maximum level. Subsequently, cell death was scrutinized by colony forming units (CFU) and flow cytometry analysis 24 h after induction (96 h after the start of the culture). As illustrated in Fig. 4B, a drastic drop of ~97% of CFU counts was found in the recombinant strain expressing the SP-lysozyme, thus supporting the efficiency of the lytic system under PHA producing conditions. In addition, we tested the contribution of 1 mM ethylenediaminetetraacetic acid (EDTA), a chelating compound that triggers membrane destabilization by reducing lipids levels, to cell lysis in the PHA-producing strains. As shown in Fig. 4B,E, there was an ~11% (CFU/ml) reduction in the wild type KT2440 strain when exposed to the chelating compound as compared to the control strain without the addition of EDTA. To fully corroborate these findings, LIVE/DEAD cell discrimination analyses were carried out using fluorescence-activated cell sorting (FACS). First, we had to discriminate between dead and live cells in the batch cultures. In order to find the gate, measured by a fluorescence signal at 678 nm, healthy bacterial cells were harvested in the middle of the exponential growth phase and examined by FACS analysis. Most of the *P*. *putida* cell population fell into the fluorescence range of 0 and 9,000 nm (Fig. 4C). Furthermore, to induce cell dead (reduction of 99.9% of cell viability), a bacterial cell population was subjected to heat-cold shock treatments, 5 cycles of freezing at −80 °C for 3 minutes followed by 3 minutes of incubation at 90 °C. The fluorescence signal for the dead cells was measured as 9,000 and 100,000 nm (Fig. 4C). The cell viability analysis showed that the wild type KT2440 strain growing on 30 (g/L) glycerol was in the spectrum range of live cells e.g. 100 to 9,000 nm (Fig. 4D). The engineered JBOi strain without induction presented a 28% of the cells with broken membrane or dead, showing similar results in comparison to CFU counts (Fig. 4B). When the lytic system was induced, 90% of the cell population presented damage in the cell membrane. The addition of EDTA accelerated the autolysis rate of cells of the JBOi strain upon induction (Fig. 4E), as well as cell mortality to 95% (Fig. 4D). To gain more insight into the dynamic cell lysis response in the recombinant *P*. *putida* strains, CFU counts were recorded following the time course of the fermentation, where toluic acid was added to the culture at 72 h of incubation (Fig. 4E). It took 10 h to observe the first decrease on CFU counts in the JBOi strain, where approximately 20% of the viable cells died each hour, leaving 2% of the total cells alive after 16 h of induction. Total PHA levels, quantified 24 h after induction (i.e. 96 h of cultivation) were maintained in the recombinant and control *P*. *putida* strains. To quantify the amount of released PHA, 5% (v/v) chloroform was poured into the culture medium for 3 h to dissolve the free PHA. It is noteworthy to mention that the amount of applied chloroform only allowed for 6% recovery of the total PHA synthesized by the wild type KT2440 strain (Fig. 4A). Therefore, the used concentration of chloroform in this study exerted a low degree of lytic activity on the cell population. More than 60% of the total PHA was recovered in the induced JBOi strain, whereas the use of EDTA improved the PHA recovery level to ~75% of the total PHA produced by the cell (Fig. 4A). The monomer composition of the produced PHAs was constant for the wild type and engineered *P*. *putida* strains. The predominant monomer was 3-hydroxydecanoate (C10), which represented ~70% of the total monomeric composition (Fig. 4F). As with the shake-flask experiments a scale-up of the recombinant JBOi strain in a 4-L bioreactor showed the same physiological patterns in terms of biomass production, PHA content and PHA recovery.

### Evaluation of cell disruption by transmission electron microscopy

**Figure 5:**
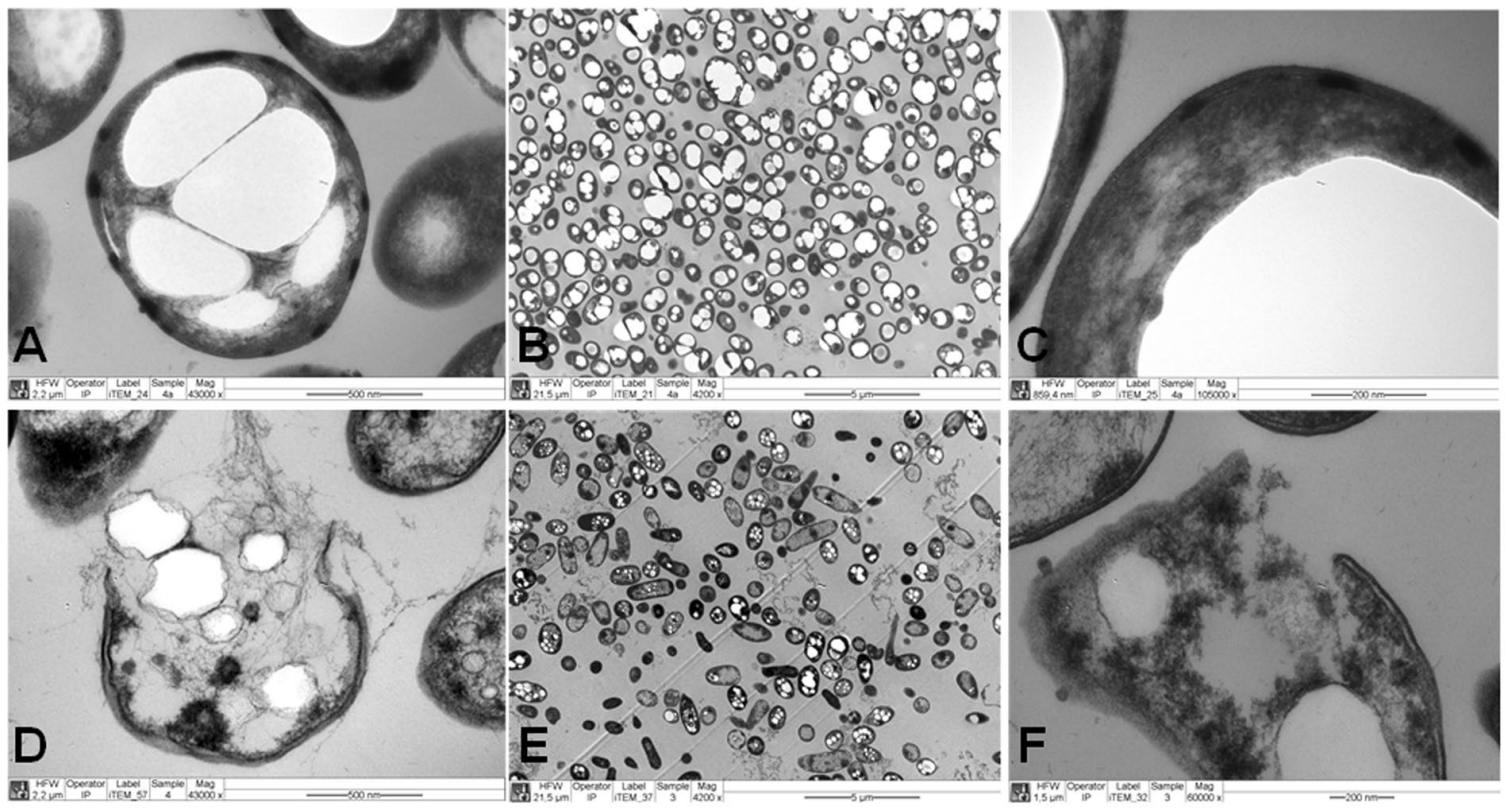
Transmission electron micrographs of *P*. *putida* KT2440 (**A**,**B**,**C**) and the engineered JBOi strain 24 h after induction (**D**,**E**,**F**). All images were taken 96 h after the start of the culture.

To gain further detail and visual insight into membrane damage in KT2440 and the JBOi recombinant strains, transmission and section electron microscopy was used on samples taken at 96 h of cultivating under PHA-producing conditions. The engineered JBOi strain showed substantial cell damage, low cell number, and release of the produced mcl-PHAs (Fig. 5D,F), whereas the wild-type strain maintained the biopolymer in the cells (Fig. 5A,B,C). The cell morphology (e.g. shape and size) of the JBOi strain also changed, resulting in larger cells as compared to the *P*. *putida* KT2440 wild-type strain (Fig. 5B,E). In addition, the morphology and number of PHA granules was also affected in the engineered JBOi strain, a feature found in many recombinant PHA-producing strains with impaired growth^28, 29^.

## Discussion

Engineered microbial strains have cost effectively advanced the sustainable synthesis of valuable chemicals and have improved the performance of bio-based processes, thereby competing with conventional manufacturing methods^30, 31^. Nevertheless, chemical compounds produced at an intracellular level, such as nanomaterials, fatty acids, and specific biopolymers, still require the use of costly mechanical, enzymatic, or chemical disruption procedures for extraction from the cell^3, 16, 32^. To address this limitation, we have created a new programmable autolysis system in the naturally PHA-producing *P*. *putida* KT2440 strain. Going beyond existing technologies for autolysis systems in cyanobacteria and bacteria (holin-endolysin), this study resulted in the generation of a new genetic construct that synthesizes the peptidoglycan-disrupting enzyme lysozyme at any desired time of the fermentation process (Table 2). Strains from the *Pseudomonas* genus are a suitable platform for antibodies and recombinant protein production^33, 34^, thus, the main basis of this study consisted in using the *P*. *stutzeri* Glucan 1,4-alpha-maltotetraohydrolase SP^25^ to influence the export of lysozyme across the inner membrane to the periplasm via the Sec pathway^35^. The oxidizing environment in the periplasmic space ensures the enzymatic catalysis of disulfide bond formation, isomerization, and the structural stabilization of enzymes^36^. First, the engineered *E*. *coli* and *P*. *putida* strain had almost the same specific growth rate and biomass yield on glycerol as the wild-type KT2440 strain under standard growth conditions. On the other hand, under PHA production conditions, a 25% reduction on biomass was observed in the recombinant JBOi strain. This demonstrates that the genetic construct exert a nutrient-dependent effect on total biomass formation of the host strain, and hence different inorganic nutrients e.g. O_2_, P, S, should be tested in the future for the synthesis of mcl-PHA using glycerol as carbon substrate. Insertion of lytic genetic circuits usually impairs growth in the microbial host strain^16, 17^. This is attributed to the basal expression of the lytic system. A possible solution to optimize the auto-lysis cell system presented in this work would be to construct genetic circuits with a tightly regulated promoter to fine-tune the basal expression of the system^37, 38^. We are currently working in improving this attractive system that, in turn, would maintain crucial physiological parameters that play key roles in the fermentation process under different nutrient limitations.

**Table 2:** Overview of various cell lysis systems and their use in biotechnological applications.

Strain	Inducible-lytic System	Phase of induction (medium)	Percentage of Dead Cells (%)	Time to achieve cell lysis (h)	Application (% product recovery)	Ref.
*B*. *megaterium*	Holin-endolysin	Stationary (glucose MM)	100	15	PHB recovery (66%)	[18]
*E*. *coli*	Holin-endolysin	Exponential (LB)	100	6	Protein synthesis	[21]
*P*. *putida* KT2440	Holin-endolysin	Exponential (Octanoate MM)	86	15	PHA recovery (16%)	[17]
*Synechocystis sp*.	Holin-endolysin	Exponential (BG-11)	100	1.5	Lipid recovery (N.D.)	[16]
*H*. *campinensis*	Holin-endolysin	Exponential (LBM)	N.S.	6	N.D.	[22]
*E*. *coli*	Holin-endolysin	Exponential (Glycerol MM)	90	48	PHA recovery (N.D.)	[19]
*E*. *coli*	Lysozyme	Exponential (Glycerol MM)	99	24	N.D.	This study
*P*. *putida* KT2440	Lysozyme	Stationary (Glycerol MM)	97	15	PHA recovery (75%)	This study

N.S. Not shown, N.T. Not determined, M.M. Minimal medium, LB. Luria Bertani medium, BG. Rich medium.

Upon induction, it took more than 10 hours to observe the first reduction in cell counts in the recombinant JBOi strain, where a 97% decrease in cell viability was achieved at 16 h post induction when cells were synthesizing mcl-PHA. This is comparable with other autolytic systems previously reported under nutrient limitations in Gram-positive and -negative bacteria (Table 2). This is highly relevant as the time used in up- and down-stream processing impacts overall costs, which could make biopolymer synthesis less competitive than conventional manufacturing procedures^39, 40^. Concerning biopolymer production and recovery, the *P*. *putida* strain harboring the genetic construct showed the same PHA accumulation — as percentage of the cell dry weight (wt/wt) — as the wild-type KT2440 strain, in addition to producing good recovery yields for the total synthesized mcl-PHA. These results were difficult to achieve in the past since the broadly employed holin-endolysin lytic system results in significantly lower values for both PHA synthesis and physiological parameters in other Gram-negative host strains^17, 18^ (Table 2). In fact, a recombinant strain of *P*. *putida* (i.e. KTHL) shows a lytic resistance phenotype attributable to the PHA granules accumulated in the cell^17^. Importantly, PHA synthesis using glucose or glycerol as carbon substrates is a non-growth associated production process in *P*. *putida* strains^9, 41^. Once nitrogen is completely consumed by the cells, PHA accumulation is initiated in the cytoplasmic space^42^. For the cell, this highly stressful environment is detrimental to the production of any heterologous protein^43, 44^. In fact, this might be the principal reason for the low cell death rates obtained using inducible lytic systems, included the one presented here, under biopolymer or fatty acid production conditions (Table 2). It is well reported that nucleic acid and protein degradation in bacterial cells occurs under all growth stages of the cell and enhanced under starvation^45–47^, amounting to 20 to 40% of degradation of the total bacterial protein content^48^. This phenomenon provides a nitrogen source to satisfy the synthesis of novel proteins involved in other metabolic processes of the cell^45, 47^. Thus, we believe that this may be the main reason that explains the synthesis of the enzymes belonging to the developed inducible cell lysis systems in bacteria even under nitrogen-limiting environments. In addition, as lysozyme is extremely effective in degrading the peptidoglycan wall of the bacterial cell^49^, a low amount of the heterologous enzyme might be required to provoke cell disruption. This was achieved in the JBOi strain, which released 62% of the synthesized mcl-PHA 24 after induction. Indeed, addition of EDTA to the culture broth allowed the produced lysozyme to act more effectively on the peptidoglycan in the cell walls^50^, thus achieving an improved release of PHA (75%) into the culture medium. Furthermore, we decided to use the pSEVA228 plasmid^26, 27^ because it can be activated with an inexpensive inducer such as m-toluic acid, which cost is about 300 times lower in comparison to the cost of Isopropyl β-D-thiogalactoside (IPTG). Another notable feature of an inducible system for lysozyme synthesis is that could be applied to a large array of PHA-producing Gram-negative bacteria, such as *Cupriavidus necator* and *Halomonas* strains, and as proven in this work for recombinant *E*. *coli* strains. These bacteria also serve as cell factories for the synthesis of biopolymers and various added-value chemicals. The promiscuity of the broad-host range plasmid pSEVA228 is another relevant factor that contributes to the feasibility of using this lytic system in other organisms. The modules responsible for replication (RK2-type origin of replication (*OriV*)^51^ and *trfA* gene)^52^, transcription (Pm promoter/*xylS* gene^53^ and strong transcriptional terminators T0 and T1)^54^ and translation (ribosome-binding site (RBS))^27, 42^ were thoroughly selected by Lorenzo and co-workers and have proven suitable for diverse bacteria^26, 55, 56^.

In conclusion, we created an effective autolysis system for cell disruption that can be activated at different growth stages during the batch fermentation process, where a 75% of the total synthesized biopolymer was recovered at the end of the fermentation period. This genetic tool can be used in multiple applications, such as protein, DNA, nanoparticles, fatty acids, and biopolymers extraction in Gram-negative bacteria. Further research should focus on the optimization of the lytic system by testing different promoters since expression was not well repressed under nitrogen-limiting conditions, thus negatively impacting on total biomass and biopolymer formation in the recombinant *P*. *putida* strain. The next step is to test the self-disrupting recombinant *P*. *putida* strain in the process of choice for industrial production PHA, the fed-batch process, which is challenging given the environmental conditions and high cell density reached in the bioreactor.

## Methods

### Genetic manipulations

The genetic system was designed with the following functional modules (5′ towards 3′): *EcoR*I restriction site, an optimized ribosome binding site (RBS; underlined) intended for strong ribosome recruitment as well as 8 conserved nucleotides (italics) AGGAGG *AAAAACAT*, the *amyP* gene 63 nucleotide leader sequence of *P*. *stutzeri* encoding for the SP amino acids MSHILRAAVLAAMLLPLPSMA ((http://www.signalpeptide.de/index.php?sess=&m=listspdb_bacteria&s=details&id=27286&listname=) & (UniProtKB - P13507 (AMT4_PSEST)), lysozyme C precursor sequence of *Gallus gallus* (NCBI: NP_990612.1) lacking its start codon and finally the *Xba*I restriction site. This sequence, termed SP-lysozyme, was synthesized by Integrated DNA Technologies, Inc., Iowa, USA and cloned into pIDTSmart plasmid which encoded an ampicillin resistance cassette. The resulting vector was named pIDTSmart-SP-lysozyme, which was introduced via transformation into *E*. *coli dam* ^−^/*dcm* ^−^ (C2925I, New England Biolabs, Ipswich, USA) as previously specified^57^. The construction of this strain leads to a fully efficient restriction after plasmid extraction since no methylation is implemented by this strain. Dam and Dcm methylation hinders *Xba*I course of action. Subsequently the *EcoR*I and *Xba*I (New England Biolabs, Ipswich, USA) digested SP-lysozyme insert was ligated into the pSEVA228 plasmid previously digested likewise. This broad-host range vector was constructed by Victor de Lorenzo and colleagues and has proven very efficient for heterologous gene expression and replication in *P*. *putida* and is inducible by toluic acid (http://wwwuser.cnb.csic.es/~seva/?page_id=17). This final construct (Fig. 1) was transferred into KT2440 strain by electroporation as described before^30^. Antibiotics were supplemented as follows: Ampicillin 100 μg/mL, for *E*. *coli*; Kanamycin 50 μg/mL for *E*. *coli* and *P*. *putida* KT2440.

### Growth conditions

*Escherichia coli* DH5α, *Pseudomonas putida* KT2440 and the recombinant JBOi and ECPi strain was kept as frozen stocks in 25% glycerol at −80 °C. To obtain single colonies, it was plated onto Luria Bertani agar plates after one day incubation at 30 °C and 37 °C, for the *P*. *putida* and *E*. *coli* strains, respectively. Inoculum was prepared by picking up a single colony from the plate and inoculating it into a 50 mL shake flask containing 10 mL of the defined minimal medium (M9) consisting of (per liter) 12.8 g Na_2_HPO_4_ · 7H_2_O, 3 g KH_2_PO4, 4.7 g (NH_4_)_2_SO_4_, 0.5 g NaCl. This medium was autoclaved and subsequently supplemented with 0.12 g of MgSO_4_ · 7H_2_O, trace elements (6.0 FeSO_4_ · 7H_2_O, 2.7 CaCO_3_, 2.0 ZnSO_4_ · H2O, 1.16 MnSO_4_ · H_2_O, 0.37 CoSO_4_ · 7H_2_O, 0.33 CuSO_4_ · 5H_2_O, 0.08 H_3_BO_3_ (mg/L) (filter-sterilized), and 4 g/L of glycerol as the unique carbon source in the *P*. *putida* strains. They were grown under aerobic conditions at 30 °C in an Ecotron incubator shaker (INFORS HT, Switzerland) set at 160 rpm. For the *E*. *coli* strains, the cells were grown at 37 °C on M9 minimal medium supplemented with 100 (µg/mL) of thiamine, 100 µM of CaCl_2_, and 4 g/L of glycerol as carbon substrate. In addition, for the recombinant strain 50 (µg/mL) of filtered Kanamycin was supplied to the medium. By taking a calculated volume of the overnight-grown cell suspension, the cells were then inoculated into 500 mL baffled Erlenmeyer flasks with 100 mL of culture medium (the same medium as described above for each bacterial strain) and cultivated in a rotary shaker. To promote PHA accumulation in the recombinant and the wild type *P*. *putida* strain, the concentration of glycerol was increased from 4 to 30 (g/L) as the only carbon and energy source in batch cultures.

### Bioreactor fermentations

*P*. *putida strains* were grown in M9 medium supplemented with 30 g/L glycerol. Bioreactor batch fermentations were carried out in a 6 L top-bench Labfor5 bioreactor (INFORS HT, Switzerland) with a working volume of 4 L, at 30 °C. The aeration rate was set to 0.5 vvm using a mass flow controller. The dissolved oxygen level was kept above 20% air saturation by control of the agitation speed up to a maximum of 700 rpm. The pH was maintained at 7.0 by automatic pH controlled addition of 0.5 M H_2_SO_4_ or 1 M of KOH.

### PHA quantification and characterization

Monomeric composition of PHA, as well as its cellular content, was determined by gas chromatography mass spectrometry (GC/MS) of the methanolyzed polyester. For this purpose, 10 mL culture broth was placed in a falcon tube and centrifuged (10 min, 4 °C, 9,000 × *g*), followed by a washing step with distilled water. The supernatant was discarded and the cell pellet was kept at −20 °C for further processing. Methanolysis was then carried out by re-suspending 5–10 mg of lyophilized aliquots in 2 mL chloroform and 2 mL methanol, containing 15% (v/v) sulfuric acid and 0.5 mg/mL 3-methylbenzoic acid as internal standard, respectively, followed by incubation at 100 °C for 4 h. After cooling to room temperature, 1 mL of demineralized water was added and the organic phase, containing the resulting methyl esters of the PHA monomers, was analyzed by GC-MS. Analysis was performed in a Varian 450GC/240MS ion trap mass spectrometer (Varian Inc., Agilent Technologies, Santa Clara, CA, USA) and operated by the software MS Workstation 6.9.3 (Varian Inc., Agilent Technologies). An aliquot (1 mL) of the organic phase was injected into the gas chromatograph at a split ratio of 1:10. Separation of compounds of interest, i.e. the methyl esters of 3-hydroxyexanoate, 3-hydroxyoctanoate, 3-hydroxydecanoate, 3-hydroxydodecanoate, 3-hydroxy-5-cis-dodecanoate, 3-hydroxytetradecanoate, was achieved by a FactorFour VF-5ms capillary column (30 m × 0.25 mm i.d. ×0.25 mm film thickness, Varian Inc., Agilent Technologies). Helium was used as carrier gas at a flow rate of 0.9 (mL/min). Injector and transfer line temperatures were 275 °C and 300 °C, respectively. The oven temperature program was: initial temperature 40 °C for 2 min, then from 40 °C up to 150 °C at a rate of 5 °C min^−1^ and finally up to 280 °C at a rate of 10 °C min^−1^. Positive ions were obtained using electron impact ionization at 70 eV and mass spectra were generated by scanning ions from *m/z* 50 to *m/z* 650. The PHA content (wt%) was defined as the percentage of the cell dry weight (CDW), represented by the polyhydroxyalkanoate.

### PHA recovery

A second set of experiments was performed to establish the PHA recovery level resulting from the JBOi induced strain. 72 mL of culture were mixed with 4 mL of pure chloroform (5% v/v final concentration). Afterwards, the sample was stirred at room temperature for 2 h and centrifuged at 3400 × *g* for 10 min at 4 °C to prevent PHA precipitation and promote chloroform-PHA phase separation from the cell material. A Pasteur pipette withdrew the chloroform-PHA phase, which was kept at room temperature until the solvent evaporated. The resulting PHA was suspended in 500 µL of chloroform and further subjected to quantification as described above.

### Colony forming unit counting

The *E*. *coli*, *P*. *putida* KT2440, ECPi and JBOi recombinant strain were grown in M9 medium supplemented either with 3 or 30 g/L glycerol in shaking flask cultures. Afterwards, the SP-lysozyme molecular system of the recombinant strain was induced with 5 mM *m*-toluic acid. Subsequently, serial dilutions of each culture were made in phosphate-buffered saline (PBS) and 100 μL were plated on LB with 2.5% w/v agar. Colony forming units (CFU) were counted on each dilution.

### FACS analysis

Flow cytometry analysis was carried out on a FACS ARIA FUSION cell sorter (BD Biosciences, San Jose CA) equipped with 2 lasers of 488 nm and 633 nm excitation wavelengths. An instrument quality control was performed by using FACS DIVA CS&T Research beads (BD Biosciences, San Jose CA) prior to bacterial cultures acquisition, according to the protocol provided by the manufacturer. In order to check for signal levels required to distinguish cells from debris that could be present on the buffers or other components from the culture media, initial tests were conducted on the flow cytometer with culture media or FACS Staining buffer (Phosphate buffered saline, Tween 20 at 0,01%) which were acquired separately to identify the noise effect, voltage and threshold settings. Bacterial cultures were stained with BD Cell Viability Kit (CAT #349483 BD Biosciences, San Jose CA) following the protocol’s instructions. Briefly, approximately 5 × 10^6^ Bacteria/mL were transferred to a 12 × 75 mm polystyrene test tube and resuspended in 200 µL of FACS Staining buffer. Control samples consisting either of unstained non-lysed bacterial cells or bacterial culture lysates subjected to heat-cold shock treatment (5 cycles of freezing at −80 °C for 3 minutes followed by 3 minutes of incubation at 90 °C) were included in order to ensure the proper settings established on the instrument, in addition to establish the gate to show the dead cells. The staining was achieved by adding 5 µL of Thiazole Orange solution 17 µM and 5 µL of Propidium Iodide solution 19 mM and mixed by reverse pipetting. Finally, after 5 minutes incubation the samples were acquired on the FACS Aria Fusion instrument.

### Transmission Electron Microscopy

Bacteria were fixed by chilling the cultures to 4 °C and addition of glutaraldehyde (2%) and formaldehyde (5%). They were then washed with cacodylate buffer (0.01 mol l) 1 cacodylate, 0.01 mol l) 1 CaCl_2_, 0.01 mol l) 1 MgCl_2_ 6H2O, 0.09 mol l) 1 sucrose, pH 6/9) and stained with 1% aqueous osmium for 1 h at room temperature. Samples were then dehydrated with a graded series of acetone (10, 30, 50, 70, 90 and 100%) with incubation for 30 min at each concentration, except for the 70% acetone, which contained 2% uranyl acetate and was performed overnight. Samples were infiltrated with an epoxy resin, according to the Spurr formula for hard resin, for several days with pure resin. Ultrathin sections were cut with a diamond knife, counterstained with uranyl acetate and lead citrate and examined in a TEM910 transmission electron micro- scope (Carl Zeiss, Oberkochen, Germany) at an acceleration voltage of 80 kV. Images were taken at calibrated magnifications using a line replica. Images were recorded digitally with a Slow-Scan CCD-Camera (ProScan, 1024 × 1024, Scheuring, Germany) with ITEM-Software (Olympus Soft Imaging Solutions, Munster, Germany). Images were recorded onto a MO-disc. Contrast and brightness were adjusted with Adobe Photoshop CS3.

### Data availability

The data that support the findings of this study are included in the article or available from the corresponding author on request.
